# Alterations of Growth Factors in Autism and Attention-Deficit/Hyperactivity Disorder

**DOI:** 10.3389/fpsyt.2017.00126

**Published:** 2017-07-13

**Authors:** Alma Y. Galvez-Contreras, Tania Campos-Ordonez, Rocio E. Gonzalez-Castaneda, Oscar Gonzalez-Perez

**Affiliations:** ^1^Department of Neuroscience, Institute of Translational Neuroscience, Centro Universitario de Ciencias de la Salud, University of Guadalajara, Guadalajara, Mexico; ^2^Unidad de Atencion en Neurosciencias, Department of Neuroscience, Centro Universitario de Ciencias de la Salud, University of Guadalajara, Guadalajara, Mexico; ^3^Laboratory of Neuroscience, School of Psychology, University of Colima, Colima, Mexico; ^4^Medical Science PhD Program, School of Medicine, University of Colima, Colima, Mexico; ^5^El Colegio de Colima, Colima, Mexico

**Keywords:** biomarker, developmental disorders, autism spectrum disorder, attention-deficit/hyperactivity disorder, growth factors, cytokines, cerebral cortex, cognitive impairment

## Abstract

Growth factors (GFs) are cytokines that regulate the neural development. Recent evidence indicates that alterations in the expression level of GFs during embryogenesis are linked to the pathophysiology and clinical manifestations of attention-deficit/hyperactivity disorder (ADHD) and autism spectrum disorders (ASD). In this concise review, we summarize the current evidence that supports the role of brain-derived neurotrophic factor, insulin-like growth factor 2, hepatocyte growth factor (HGF), glial-derived neurotrophic factor, nerve growth factor, neurotrophins 3 and 4, and epidermal growth factor in the pathogenesis of ADHD and ASD. We also highlight the potential use of these GFs as clinical markers for diagnosis and prognosis of these neurodevelopmental disorders.

## Introduction

During neural development, a myriad of biological events occurs simultaneously, i.e., neurogenesis, gliogenesis, cellular migration, cell differentiation, synapse formation, etc. These neurobiological processes are orchestrated by several growth factors (GFs) and help shape the postnatal brain (1). In the postnatal brain, GFs have been extensively studied and most of them share similar cell functions to those reported in the neurodevelopment (2–4). Increasing evidence indicates that GFs modulate motor, emotional, and cognitive functions, which may explain several clinical manifestations of psychiatric disorders (5).

Neurodevelopmental disorders comprise a group of neurological conditions that are considered a public health problem with strong socioeconomic impact (6). These disorders have a very complex etiology and are characterized by early-onset during childhood. The initial pathological change appears to involve abnormal growth-factor expression during embryogenesis, which persist in the adulthood and may contribute to some clinical manifestations (6).

Some of the most common neurodevelopment disorders are autism spectrum disorder (ASD) and attention-deficit/hyperactivity disorder (ADHD). These disorders generate a poor global performance throughout life. ADHD and ASD have a complex etiology and their pathophysiology remains unclear. ADHD and ASD are often comorbid disorders (7) that share some morphological, molecular, and functional characteristics, such as: abnormal growth of neural tissue (8), cognitive impairment (9), male preponderance, epigenetic components (8, 10), and abnormal expression levels of GFs in serum and brain. Remarkably, the expression level of some growth-factor correlates with the clinical manifestations of ADHD and ASD (11). This evidence suggests the role of GFs in the pathophysiology of these disorders. Herein, we summarize the current evidence obtained in humans and animal models that associate GFs levels with ADHD and ASD (Table 1). This correlation unveils the attractive possibility to use GFs as serological biomarkers to establish diagnosis and prognosis for these disorders.

**Table 1:** Relationship between growth-factor levels and clinical symptoms in neurodevelopmental disorders.

Neurodevelopment disorder	Growth factor	Population/animal model	Biological sample analyzed	Related symptoms	Genetic polymorphism
Attention-deficit/hyperactivity disorder	Brain-derived neurotrophic factor (BDNF) ↓ (12–14)	Children and adolescents (14)	Blood sample	Hyperactivity (15)	BDNF (rs10835210 and rs11030101) (16, 17)
		Adult (12)	Blood sample	Impairment of spatial learning (13)	BDNF (rs6265/Val) (18)
		Adult male spontaneous hypertensive rats (SHR) (13)	Hippocampus (13)		
		BDNF^2lox/2lox^/93 mice (19)	Hippocampus, hypothalamus, and cortex (19)		
		Dopamine transporter knockout mice (DAT^−/−^) (20)	Frontal cortex (20)		

	Glial-derived neurotrophic factor ↑ (11, 21)	Children (21)	Blood sample	Inattention, hyperactivity and impulsivity behaviors (11)	Undefined

	Nerve growth factor (NGF) ↑ (22, 23)	Children and adolescents (23)	Blood sample	Attentional, learning and memory impairments (24, 25)	NGF (rs6330) (24)
		Adult male SHR (22)	Blood sample		

	Neurotrophin-3 ↑ (21)	Children (21)	Blood sample	Undefined	Undefined

	Vascular endothelial growth factor ↓ (26, 27)	Juvenile male stroke-prone spontaneously hypertensive rats (SHRSP) (26, 27)	Frontal cortex (27)	Undefined	Undefined

	Insulin-like growth factor 2 ↑ (28)	Children (28)	Blood sample	Undefined	Undefined

	FGFR1 ↓ (29)	*Fgfr1*^f/f;hGfapCre^ mice	Dorsal telencephalon (29)	Spontaneous motor hyperactivity (29)	Undefined

Autism spectrum disorder	TGF-β1 ↓ (30)	Children (30)	Blood sample	Low adaptive behaviors, stereotypy, irritability and low social interaction (30)	Undefined

	Epidermal growth factor ↓ (31, 32)	Adult (31)	Blood sample	Hyperactivity, deficit in gross motor skills, tendency for tip toeing (32)	Undefined
		Children (32)	Blood sample		

	BDNF ↑ (33, 34)	Valproic acid (VPA)-treated rat offspring (33)	Hippocampus (33)	Undefined	Undefined
		Children (34)	Blood sample		

	Neurotrophin-4 ↓ (35)	Children (35)	Blood sample	Undefined	Undefined

	Hepatocyte growth factor ↓ (36)	Children (36)	Blood sample	Undefined	Undefined

*Arrows represent the level variation: increased expression (upward position) and reduced expression (downward position)*.

## Attention-Deficit/Hyperactivity Disorder

The ADHD has the highest incidence rate among all neurodevelopmental disorders (37). ADHD is characterized by inappropriate levels of inattention, hyperactivity, and impulsivity (15). These patients have evident social and academic problems that affect their global performance (38, 39). During the childhood, the main manifestation of ADHD is hyperactivity, which is commonly identified at the preschool, whereas inattention becomes more evident at elementary school (7). Children with ADHD also present negative emotionality, high emotional lability, and poor emotion management (39). During adolescence, patients with ADHD have high risk to suffer motor vehicle accidents, spontaneous sexual encounters, sexual diseases, unwanted pregnancies, drug abuse, poor social relationship, legal problems, etc. (37). Some of these ADHD symptoms persist until adulthood (37). In adults, ADHD induces a predisposition toward deficient relationships, substandard job performance, low socioeconomic status, and poor quality of life (37). ADHD is highly comorbid with other psychiatric or neurodevelopment disorders, such as oppositional defiant disorder, major depressive disorder, and anxiety disorders (37, 40).

Catecholamine dysfunction is the main hypothesis to explain ADHD pathophysiology; specifically, the dysfunction in dopamine receptors D4, D5, and in dopamine transporter proteins (15, 41) in prefrontal cortex, nucleus accumbens, striatum, substantial nigra, ventral tegmentum, and frontal cortex (6). The homeostasis of dopamine system requires the brain-derived neurotrophic factor (BDNF), a widely expressed neurotrophin in brain cortex and hippocampus (42). BDNF is critical in the synthesis, release, and uptake of dopamine in nigro-striatal dopaminergic neurons (43, 44) and plays a fundamental role in neuronal survival, plasticity, and proliferation (12). During development, BDNF and its receptors TrkB not only promote survival and differentiation of neurons but also are involved in neural plasticity in adulthood (13). Alteration in BDNF/TrkB activity is implicated in midbrain dopaminergic dysfunction reported in ADHD, which may explain the development of the main symptoms (45). Low serum levels of BDNF in ADHD can persist until adulthood (12). This indicates that BDNF signaling alteration occurs across life spam in patients with ADHD.

Polymorphisms rs11030101 and rs10835210 have been associated with a high risk to develop ADHD (16, 17). Some BDNF polymorphism are related to gender. The BDNF polymorphism rs6265/Val is more frequent in women with ADHD (18). This polymorphism has also been associated with susceptibility to neuroticism and anxiety (46), which may explain some psychiatry comorbid disorders. Conditional BDNF mice (BDNF^2lox/2lox^/93), characterized by low BDNF expression in the hippocampus, hypothalamus, and cortex, develop hyperactivity and aggression after stress (19), which supports the role of BDNF in the processing of motor control or in the worsening of hyperactivity symptoms of ADHD.

The role of BDNF in ADHD pathophysiology is not fully understood, but the evidence in animal research provides clues to understand the biochemical mechanism that underlie this condition. These patients show several alterations in cognitive process and memory performance (47) that may be due to hypoactivation of prefrontal cortex (48). In the spontaneous hypertensive rats, an animal model for ADHD, have been found low levels of BDNF and TrkB in the hippocampus that were related to memory impairment (13). These findings suggest that cognitive manifestations of ADHD might be associated with alterations in BDNF signaling. In dopamine transporter knockout mice (DAT^−/−^), another animal model for ADHD, low expression level of BDNF mRNA and TrkB receptors were found in frontal cortex (20). BDNF regulates two crucial circuits, the fronto-striatal-cerebellar and the ventral striatal-limbic circuits in normal brain (45). Neural circuits in prefrontal cortex and cerebellum, which modulate the attentional process, thoughts, emotions, social behavior, and motor control, are implicated in ADHD symptomatology (37).

Patients with ADHD show ~5% reduction in brain volume (15) in several regions, such as corpus callosum, orbitofrontal cortex, hippocampus, amygdala, basal ganglia, temporal lobe, prefrontal cortex, caudate, and cerebellum (49). Low levels in BDNF expression may explain this volume reduction, as demonstrated in late-onset forebrain-specific BDNF knockout (CaMK-BDNF^KO^) mice (50). On the other hand, patients with the pure form of ADHD lack microstructural changes in white matter tracts (40). In this study, the authors associated these microstructural changes with the clinical manifestations of ADHD and reported that the brain volume in gray and white matter correlates to poor cognitive processing, attention and motor planning (37). Interestingly, BDNF-deficient (bdnf^−/−^) mice show a significant reduction in myelin proteolipid protein and myelin basic protein in the hippocampus and cortex, with a subsequent deficit in myelination (15). Altogether, these data support the hypothesis that the BDNF signaling pathway is associated with changes of cognitive performance and brain structure in ADHD. Several pharmacological treatments that modulate the symptoms of ADHD can also modify GFs levels. Tricyclic antidepressants and the selective serotonin reuptake inhibitors increase the levels of BDNF (15). Methylphenidate, the main drug prescribed for ADHD, increases the BDNF expression in the prefrontal cortex (51, 52). A 6-week administration of methylphenidate recuperates the plasma levels of BDNF in children with ADHD (42).

Glial-derived neurotrophic factor (GDNF) is another GF-related to the pathophysiology of ADHD. GDNF is widely involved in the survival of serotonergic and dopaminergic neurons because it has neuroprotective effects against neuroinflammation and oxidative damage (21). Untreated children with ADHD show high plasma levels of GDNF (21). These high GDNF levels have a positive correlation with inattention, hyperactivity, and impulsivity behaviors (11), which are the main clinical manifestations of ADHD. Remarkably, psychostimulants, such as MPH, increase levels of GDNF mRNA in the hippocampus and prefrontal cortex (51). Furthermore, nerve growth factor (NGF) is involved in neuronal development and brain plasticity of cholinergic neurons that are important in attentional processing. Therefore, dysregulation of NGF levels have been associated with the pathophysiology of ADHD (23). At genetic levels, the single nucleotide polymorphism (rs6330) is associated with the risk of ADHD (24). High NGF levels are found in an animal model of ADHD (22). Interestingly, children and adolescents with ADHD show high NGF serum levels (23). These alterations in the pro-NGF and/or NGF levels are related to attentional, learning, and memory impairments shown by ADHD patients (24, 25). Neurotrophins also play a role in the pathophysiology of ADHD (21). Alterations in neurotrophin-3 (NTF3) expression are considered a risk factor for ADHD in childhood (2). Serum NTF3 levels are increased in untreated patients (21).

Vascular endothelial growth factor (VEGF) has an important role during brain development and repair (27). In stroke-prone spontaneously hypertensive rats were found downregulation of VEGF (26, 27), VEGFR-1 (Flt-1), and VEGFR-2 (Flk-1) receptors, endothelial nitric oxide synthase and the phosphorylated Akt in frontal cortex (26, 27). Since alterations on VEGF signaling have been associated with degeneration of cerebral cortex, it is possible that these alterations are implicated in cerebral abnormalities of patients with ADHD (26, 27).

Insulin-like growth factor 2 (IGF2) regulates normal development of cerebellum and hippocampus (50, 53), both of which are affected in ADHD (43, 44). Recently, IGF2 DNA methylation may be as predisposing factor to develop ADHD (33, 54, 55). In fact, prenatal exposure to high-fat and -sugar diet promotes IGF2 DNA methylation at birth that, in turn, has been positively associated with higher ADHD symptoms (28). Another GF that has also been implicated in hyperactive behavior include the fibroblast growth factor. In rodents, disruption of the Fgfr1 gene in dorsal telencephalon causes spontaneous motor hyperactivity and significant reductions in specific types of cortical inhibitory neurons (29). In humans, the role of FGFR in the etiology of ADHD has been suggested by pathway analysis of FGFR1b and FGFR2b activation, but further study is needed to support this assertion (56). In summary, the development of ADHD may be influenced by the interaction of multiple molecules and concomitant epigenetic factors, but confirmatory studies are required to reveal more definitive associations.

## Autism Spectrum Disorder

Autism spectrum disorder is a neurodevelopmental disorder (35, 57) that is typically diagnosed between 2 and 6 years of age (8). ASD is characterized by impaired social communication, repetitive, or stereotyped behaviors and low interest for environment stimuli (7, 58). Patients with ASD have maladjustment in emotional response, anxiety, impaired emotional learning, limited interest in surrounding environment, and deficit in communication and social interactions (32, 59). ASD pathology has several associated symptoms that are generated by comorbid disorders. These comorbid symptoms often include: seizures, anxiety, intellectual impairment, hyperactivity, hyper or hypo- responsiveness to stimulus, sleep disruption, aggressive behavior, etc. (8). Consequently, ASD is considered a complex disorder with important epigenetic components (8, 60, 61).

Acetylation is a common feature of the neurotrophic proteins encoded by at least 18 genes dysregulated in patients with ASD (62, 63). While there is some evidence of the role of immune system dysregulation in the etiology of autism (64), it is possible that acetylation, lysine methylation/demethylation of histones, and inflammatory mediators affect mutual signaling pathways in both the nervous system and the immune system (65). Increasing evidence suggests that the abnormal increase of brain cortex and minicolumnar abnormalities observed in autism are driven by excess neuronal production (66–68). This hypothesis has been supported by neuroimaging studies (69–71) and three-dimensional neural cultures (a cerebral organoid model) with induced pluripotent stem cells (72). Since GFs regulate different aspects of neural development, including brain growth, stem cell proliferation, and cell survival, this evidence supports their role in the development of ASD (32) and may explain the enlargement of prefrontal and temporal cortex that persists until adulthood (73). The hyper-functioning in certain neural circuits observed in autism may be due to this uncontrolled growth of neural connections (35, 57, 74).

The brain volume is considered as clinical indicator of certain psychiatry disorder (75). In patients with ASD, the brain enlargement and abnormal neuronal migration have been observed in regions, such as the subependymal cell layer, the granule layer in the dentate gyrus, the cornu ammonis subfield, and the amygdala (76). In contrast to this abnormal migration and brain overgrowth, some authors have reported a low rate of growth in other brain areas (77). In the post-mortem brain of patients with ASD has been observed a significant reduction of neuronal density in layer III, the total number of neuron in layer III, V and VI, and a decrease in the volume of neurons in layers V and VI at the fusiform gyrus (77). Another study reported a decrease in pyramidal neuron size in the inferior frontal cortex, specifically in the Brodmann’s areas 44 and 45, which are brain regions involved in language processing, imitation function, and sociality processing (78).

Children with autism show low expression levels of Neurotrophin-4 (NTF4) in blood, which have been correlated to impairments in neuroplasticity (35). TGF-β1 plasma levels are also reduced in autism and have a significant correlation with the low scores obtained in adaptive behaviors, stereotypy, irritability, and low social interaction (30). Similar findings have been reported in juvenile mice in which the treatment with TGF-β1 impairs social interaction and promotes repetitive and stereotyped behaviors. Intriguingly, TGF-β1 overexpression has the opposite effects in adult stages (79).

Brain-derived neurotrophic factor serum levels are significantly increased in autism (34). High expression of BDNF is also found in a model of autism [valproic acid (VPA)-treated rat offspring] (54). In addition to the high BDNF expression in hippocampus, the VPA-treated rats show low expression of p-Akt, Bcl-2, p-CaMKII, as well as a significant increase in Bax and caspase-3 expression (33). This evidence is consistent with that found in BTBR T+tf/J mice (another autistic mouse strain), which present a significant upregulation of BDNF expression and myelin protein, and low expression of glial fibrillary acidic protein (55).

Another GF involved in the pathophysiology of ASD is the epidermal growth factor (EGF). EGF strongly promotes cell proliferation and differentiation *via* MAPK, PKC, and Akt pathways in neural progenitor cells (4). Recently, EGF and its receptor protein (EGFR) have been proposed as biomarkers of schizophrenia, depression, and bipolar disorder [reviewed in Ref. (5)]. Interestingly, adult patients with high-functioning ASD show a significant reduction in serum levels of EGF as compared with controls (31). Similar findings are observed in children with ASD (32), who present persistent low plasma levels of EGF until adulthood (80). Remarkably, these low plasma levels negatively correlates with the severity of hyperactivity, the deficit in gross motor skills, and the tendency for tip toeing (32). In addition, patients with ASD show a reduction in Akt phosphorylation, EGFR overexpression and low levels of gamma-aminobutyric acid that correlate to the severity of alterations in several language components (81). Elevated Akt phosphorylation has also been found in prenatal exposure to valproate, a well-known animal model of autism (82). These animals show a significant growth of several brain regions, a common alteration that is also observed in autistic patients. Taken together, these data strongly support the notion that EGFR/AKT pathway may play an important role in the pathophysiology of ASD.

Hepatocyte growth factor (HGF) is another molecule that has been involved in the development of ASD (36, 83). Although low levels of HGF have been reported in patients with ASD, these findings could not be correlated to the severity of symptoms (36). HGF promotes morphogenesis and cell proliferation after binding to the c-Met receptor, a product of the MET gene (84). Interestingly, polymorphisms in the MET gene appear to confer an increased susceptibility to autism and this gene is included in the chromosome 7q31 that has been linked to autism susceptibility (85, 86). This evidence suggests that HGF may represent an important factor in the pathogenesis of autism.

## Future Approaches in Neurodevelopmental Disorders

**Figure 1:**
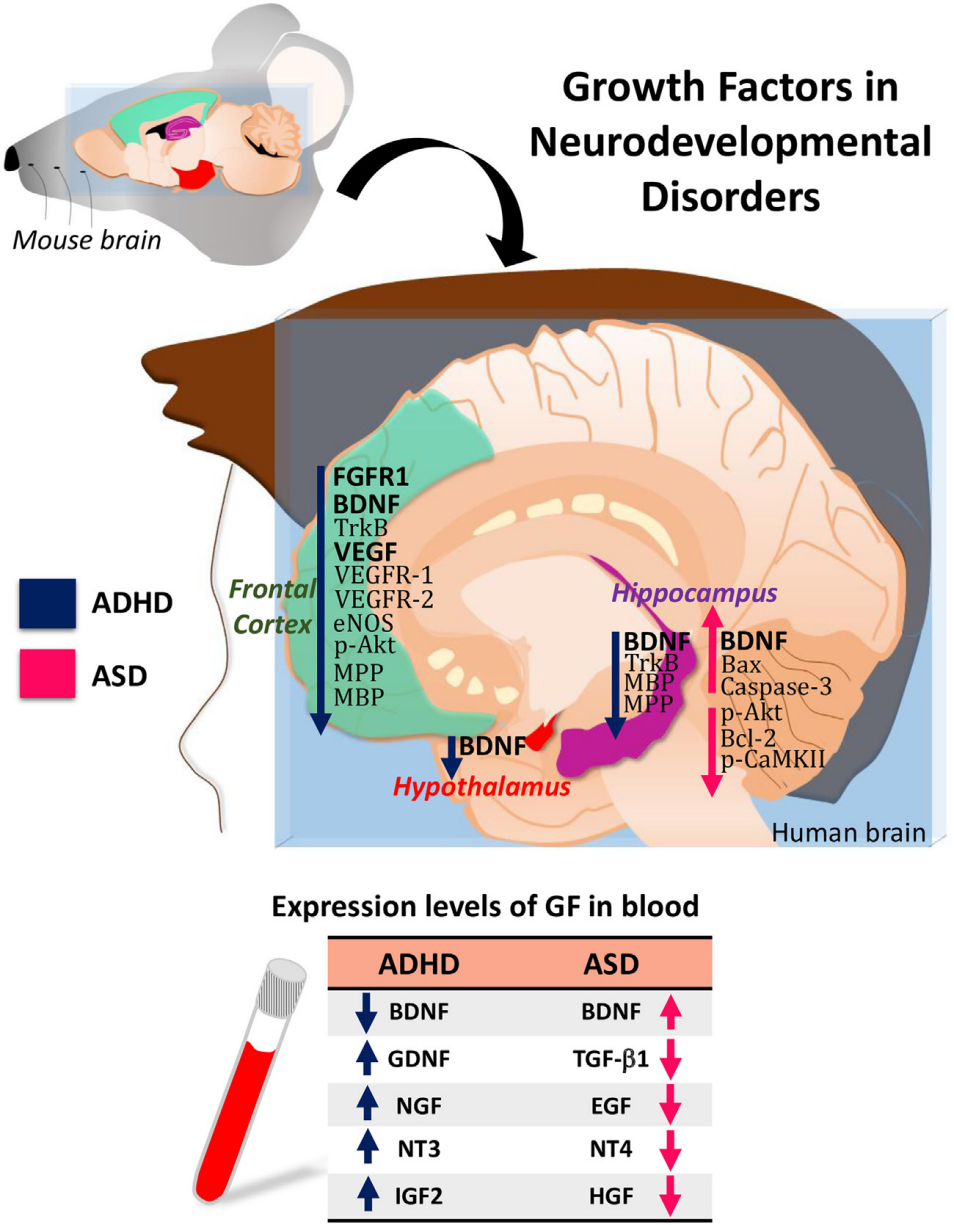
Hypothetical model of growth-factor expression in the human brain based on data obtained in attention-deficit/hyperactivity disorder (ADHD)/autism spectrum disorder (ASD) animal models. The table shows the blood levels of growth factors (GF) found in patients with ADHD or ASD.

Attention-deficit/hyperactivity disorder and ASD are neurodevelopmental disorders with significant comorbidity. Increasing evidence indicates that they share some pathological features and some etiological factors. Alterations in the expression level of BDNF, GDNF, NGF, NTF3, NTF4, or EGF are common features between ADHD and ASD (Figure 1). Therefore, identifying all these aspects will allow to establish the clinical use of these GFs as biomarkers in ADHD and ASD.

## Author Contributions

AG-C contributed in the conception idea for the article, in the manuscript writing, and in the table design. TC-O participated in the manuscript writing and in the table and the figure design. RG-C participated in the manuscript writing and in the table and figure design. OG-P contributed to the conception of the idea for the article and in the manuscript writing type.

## Conflict of Interest Statement

The authors declare that the research was conducted in the absence of any commercial or financial relationships that could be construed as a potential conflict of interest.
